# Skin color influences transcutaneous bilirubin measurements: a systematic in vitro evaluation

**DOI:** 10.1038/s41390-024-03081-y

**Published:** 2024-02-17

**Authors:** Alida J. Dam-Vervloet, Claudia F. Morsink, Marleen E. Krommendijk, Ingrid M. Nijholt, Henrica L. M. van Straaten, Lieke Poot, Nienke Bosschaart

**Affiliations:** 1 Biomedical Photonic Imaging group, Technical Medical Centre, University of Twente, Enschede, The Netherlands; 2 Medical Physics Department, Isala hospital, Zwolle, The Netherlands; 3 Innovation & Science Department, Isala hospital, Zwolle, The Netherlands; 4 Radiology Department, Isala hospital, Zwolle, The Netherlands; 5 Neonatology Department, Isala hospital, Zwolle, The Netherlands

## Abstract

**Objective:**

Concerns have been raised about the effect of skin color on the accuracy of transcutaneous bilirubin (TcB) measurements, a widely used method for hyperbilirubinemia diagnosis in newborns. Literature is inconclusive, with both reported under- and overestimations of the TcB with increasing skin pigmentation. Therefore, the influence of skin color on TcB measurements was systematically evaluated in a controlled, in vitro setting.

**Methods:**

A bilirubin meter (JM-105) was evaluated on layered phantoms that mimic neonatal skin with varying dermal bilirubin concentrations (0–250 µmol/L) and varying epidermal melanosome volume fractions (0–40%; light-dark skin color).

**Results:**

TcB measurements were influenced by skin pigmentation. Larger mimicked melanosome volume fractions and higher bilirubin levels led to larger underestimations of the measured TcB, compared to an unpigmented epidermis. In the in vitro setting of this study, these underestimations amounted to 26–132 µmol/L at a TcB level of 250 µmol/L.

**Conclusion:**

This in vitro study provides insight into the effect of skin color on TcB measurements: the TcB is underestimated as skin pigmentation increases and this effect becomes more pronounced at higher bilirubin levels. Our results highlight the need for improved TcB meter design and cautious interpretation of TcB readings on newborns with dark skin.

**Impact:**

- Key message: Skin color influences transcutaneous bilirubin measurements: the darker the skin, the larger the underestimation.
- What this study adds to existing literature: Existing literature is inconclusive regarding the influence of skin color on transcutaneous bilirubin measurements. This study systematically evaluates and clarifies the influence of skin color on transcutaneous bilirubin measurements in a controlled, in vitro setting.
- Impact: This study aids to better interpret the measured TcB level in patients with varying skin colors, and is particularly important when using TcB meters on patients with dark skin colors.

## Introduction

**Fig. 1:**
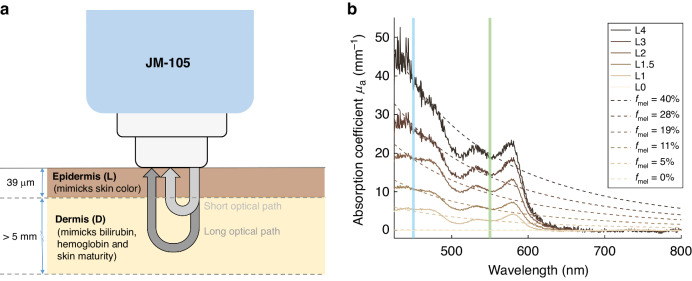
TcB meter and phantom absorption spectra of neonatal epidermal phantom layers. **a** Schematic drawing of the evaluated TcB meter (JM-105) and a phantom with an epidermal and dermal layer. In this study, different combinations of a single epidermal, and dermal phantom layer were used to mimic variations in both skin color and TcB. **b** Measured absorption spectra (solid lines) of the epidermal phantom layers L0-L4, with mimicked melanosome volume fractions f_mel_ ranging from 0 to 40% (figure adapted from ref. ^31^). At wavelengths of 450 nm (blue) and 550 nm (green) evaluated by the transcutaneous bilirubin (TcB) meter, the absorption coefficients of the epidermal phantom layer were in good agreement with the reference spectra from literature^44^ (dashed lines).

Jaundice in newborn infants is a common clinical condition, affecting up to 80% of infants in the first week after birth.^1,2^ Severe jaundice, or hyperbilirubinemia, can lead to Bilirubin Induced Neurologic Dysfunction (BIND), which can cause irreversible brain damage.^3^ Therefore, international guidelines recommend screening of newborn infants at risk for hyperbilirubinemia.^4,5^ Since 1980, transcutaneous bilirubinometry has become an effective noninvasive screening method for hyperbilirubinemia in newborn infants. It is superior to visual inspection of the newborn infant and can be used to reduce the number of invasive total serum bilirubin (TSB) determinations.^6–12^ Transcutaneous measurements cannot completely replace TSB determinations, as the transcutaneous bilirubin concentration (TcB) is a physiologically different parameter from the TSB.^10^ TcB meters are based on optical spectroscopy and relate the absorption around the bilirubin absorption peak at 450 nm to its concentration.^9^ Commonly used TcB meters (JM-103 and JM-105) use a second wavelength at 550 nm to correct for the background absorption of hemoglobin.^13,14^ These types of TcB meters also use a short and a long optical path to collect backscattered light (Fig. 1a). The manufacturer states that the difference between these two paths is used to correct for background absorption of melanin in the epidermal layer (i.e. skin color).^13,14^ Nevertheless, many in vivo studies report on the effect of skin color on TcB measurements.^15–25^ These studies are inconclusive regarding the exact effect of skin color. Some studies suggest that the TcB overestimates the TSB in newborn infants with a darker skin color,^15–19,24^ up to an average of 52 µmol/L,^24^ whereas other studies report an average underestimation^17,20–23,25^ up to 19 µmol/L.^23^ These inconclusive results may be explained by the large variation in patient populations between these studies, as other patient factors (i.e. skin thickness, skin maturity and body location) may also significantly influence TcB measurements.^26,27^ In addition, the low inter-device reproducibility of the TcB meter may have played a role.^28^

TcB meters are not the only optical devices to suffer from an inadvertent bias towards darker skin colors. Optical pulse oximeters that are routinely used in intensive care, were recently found to overestimate oxygen saturation in patients with darker skin colors.^29,30^ This has significant implications for patient care: the diagnosis of hypoxemia is three times more likely to be missed in darker-skinned patients than in lighter-skinned patients.^29,30^

Also for TcB meters, the impact of skin color on patient care should not be underestimated. In particular an underestimation of the TcB can potentially have a negative impact on the management of hyperbilirubinemia, by delaying the decision for follow-up TSB determination and to start treatment with phototherapy.

Ideally, to obtain unambiguous evidence on the effect of skin color on TcB measurements, all other sources of variation should be eliminated. As this is practically impossible in an in vivo patient setting, we designed an in vitro study to systematically evaluate the influence of skin color on TcB determinations. This study builds on our previous work on the design of dedicated phantoms for TcB meter evaluation^26,28^ and introduces a new approach to incorporate mimicked epidermal melanin layers into these phantoms.^29^ We evaluated a bilirubin meter (JM-105) on phantoms that mimic neonatal skin with varying bilirubin concentrations (0–250 µmol/L) and varying epidermal melanosome volume fractions (0–40%; light – dark skin color).

## Materials and methods

### TcB-meter

TcB measurements were performed using the transcutaneous meter JM-105 (serial number: B3601137, Dräger Medical, Lübeck, Germany), which is widely used in pediatric departments in the Netherlands. The accuracy of the TcB measurements specified by the manufacturer is 25.5 μmol/L (>35 weeks GA) and 27.4 μmol/L (>24 weeks GA).^14^ A single TcB meter was used throughout this study, to avoid the potential influence of low inter-device reproducibility.^28^

### Neonatal skin-mimicking phantoms

The influence of skin color on TcB measurements was evaluated using a series of neonatal skin-mimicking phantoms. Phantoms are generally used to mimic tissue properties in a controlled manner, allowing systematic evaluation of the performance of medical devices. The phantoms that were designed for this study consist of a thin epidermal phantom layer and a dermal phantom layer (Fig. 1b). These phantom layers both accurately simulate (the variation in) the optical absorption and scattering properties of neonatal skin at the two wavelengths that are evaluated by the TcB meter (450 and 550 nm).

#### Epidermal phantom layer

Light absorption by melanin in the epidermal layer was mimicked using a series of thin phantom layers. The systematic design and characterization of this specific series of phantom layers has been described in detail in our previous work.^31^ In summary, thin epidermal phantom layers were prepared by color printing on adhesive polyurethane film dressings (Tegaderm^TM^, 1634W, 3M Healthcare) using a standard office laser printer (Xerox 7800 series, type WorkCentre 7835i) with magenta and yellow toner (Xerox WW metered code 006R01511 and 006R01510, respectively). As the optical absorption coefficient of these printed toner colors on polyurethane scales linearly with print opacity (0% = no color, 100% = full color), the printing process can be designed such that the resulting phantom layer absorption matches the desired epidermal absorption for a range melanosome volume fractions.^31^ Input settings for printing and communication with the printer were achieved using the open-source graphics software InkScape (version 1.2).

The target melanosome volume fraction (*f*_*mel*_) and corresponding optical absorption coefficients for the epidermal phantom layers were derived from literature and ranged from albino (0%), to light skin (1.3–6.3%), to moderately pigmented skin (11–16%), to darkly pigmented skin (18–43%).^32^ Table 1 lists the six epidermal phantoms (L0-L4), with their mimicked melanosome volume fractions and their corresponding absorption coefficients at 450 nm and 550 nm. These absorption coefficients were measured in collimated transmission, as described in our previous work.^31^ Fig. 1b shows that the mimicked (dashed lines) and measured (solid lines) absorption coefficients at 450 nm and 550 nm - the two wavelengths at which the TcB meter operates - were in good agreement.

**Table 1:** Specification of epidermal (L0- L4) and dermal (D1- D9) phantom layer properties.

Epidermal phantom layer			
Epidermal layer^a^	Mimicked melanosome volume fraction (%)	Measured µ_a_ 450 nm (mm^−1^)	Measured µ_a_ 550 nm (mm^−1^)
L0	0	0	0
L1	5	5.5	2.7
L1.5	11	10.7	5.7
L2	19	18.9	10.6
L3	28	26.4	15.0
L4	40	39.6	19.7
**Dermal phantom layer**			
Dermal layer	Mimicked TcB^b,c^ (µmol/L)	Measured µ_a_ 450 nm (mm^−1^)	Measured µ_a_ 550 nm (mm^−1^)
D1	0	0.50	0.32
D2	22	0.65	0.32
D3	87	1.09	0.32
D4	145	1.74	0.33
D5	172	2.21	0.33
D6	198	2.70	0.33
D7	222	3.25	0.34
D8	238	3.97	0.34
D9	249	4.49	0.35

Epidermal phantoms mimicked a series of melanosome volume fractions (%). Dermal phantom layers mimicked a series of TcB levels (µmol/L).

^a^The print opacity was set at 60% for magenta and 22% for yellow, which mimicked the target optical absorption of melanin at 450 nm and 550 nm. The absorption coefficient was increased by running a film multiple times through the printer with the same print assignment, i.e. by varying the number of printed toner layers from 0 to 4.^31^

^b^Mimicked TcB corresponds to the average measured TcB through a plain Tegaderm^TM^ film (L0).

^c^Conversion factor 1 µmol/L = 0.058 mg/dL bilirubin.

The thickness of the epidermal layer phantom was measured by optical coherence tomography to be 39.2 ± 3 μm (average ± standard deviation),^31^ which is comparable to the thickness of the neonatal epidermis.^33^

#### Dermal layer

The influence of skin color was evaluated on nine aqueous dermal phantoms (D1–D9, Table 1), that accurately mimic the optical absorption and scattering properties of neonatal skin over the clinically relevant bilirubin concentration range (0 to 249 μmol/L). The dermal layers were prepared according to the procedure of our previous work.^26,28^ The target optical properties of the skin phantom layers were derived from an in vivo study on the optical properties of neonatal skin.^34^

Briefly, skin absorption by hemoglobin and bilirubin was mimicked by two dyes (Ecoline: Magenta-337 and Light-Yellow-201, Royal Thalens, The Netherlands). The mimicked TcB was varied by adjusting the individual concentrations of both dyes, which changed the optical absorption around 450 nm and 550 nm (Table 1). The absorption coefficient at 550 nm was kept constant to mimic a stable cutaneous hemoglobin concentration of 2.13 g/L, which is the average value for neonatal skin.^34^ Light scattering was mimicked by adding a dilution of the standard tissue scattering phantom Intralipid (Intralipid® 20%, Fresenius Kabi, Bad Homburg, Germany), resulting in a reduced scattering coefficient (µ_s_^’^) of 2.00 mm^−1^ at 450 nm and 1.63 mm^−1^ at 550 nm, which is the average value for neonatal skin.^34^ The measured TcB on the dermal phantom D4 through the unprinted epidermal phantom L0 exhibited a minimal drift as a function of time throughout the duration of the experiments (~7 h), which was well-predictable (R^2^ = 0.96) with the formula: TcB(*t*) = TcB(*t* = 0) + 0.044t, where *t* represents time in minutes. All TcB measurements were corrected for this minimal drift.

### Experimental set-up

The same experimental set-up was used as in our previous studies.^26,28^ In summary, to ensure that the measurement tip of the TcB meter could be pressed down, which is a prerequisite for correct operation, the neonatal skin mimicking phantoms were covered by a rigid steel plate with an opening (Ø 8.8 mm) to accommodate the TcB meter (outer optical detection ring Ø 8.1 mm).

In the set-up, the tip of the TcB meter was covered by the epidermal phantom layer, which was then positioned on top of the dermal phantom layer to establish direct contact between the two layers, similar to the geometry in Fig. 1a. Air bubbles adhering to the bottom of the dermal layer were removed by gentle scraping with a rubber block, prior to the measurement.

For all combinations of epidermal and dermal phantom layers, the TcB was measured as the average ± standard deviation (SD) of five measurements to obtain a good estimate of the variation in our data. With six epidermal and nine dermal phantom layers, this resulted in a total of 5 × 6 × 9  =  270 phantom measurements.

## Results

**Fig. 2:**
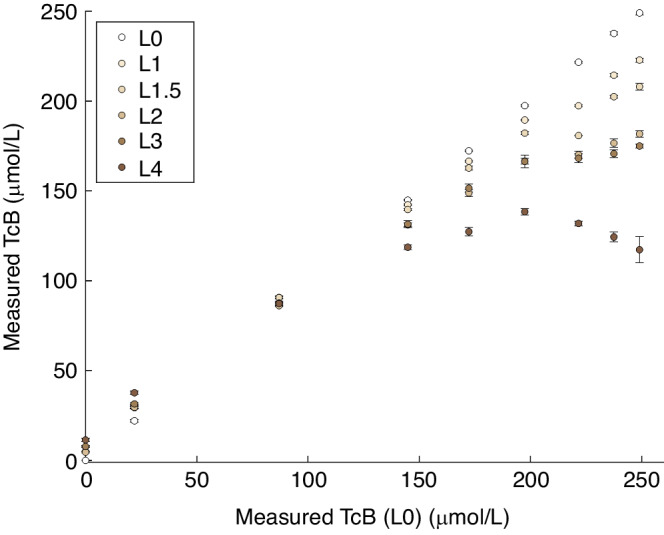
Effect of skin color on TcB measurements. Measured TcB levels per epidermal phantom layer (L0–L4, mimicked melanosome volume fractions ranging from 0–40%, Table 1) on nine dermal layers (D1–D9; mimicked TcB ranging from 0–249 μmol/L, Table 1) as a function of measured TcB through epidermal phantom layer L0 (mimicked melanosome fraction 0%). Error bars represent the standard deviation of five TcB measurements per phantom.

Figure 2 shows the effect of skin color on the TcB measurements. Adequate performance of the TcB meter would ideally result in the same TcB level for each mimicked skin color over the entire TcB range. However, we observed a significant dependence of the measured TcB on skin color (Fig. 2). The measured deviation in TcB increased with increasing skin color and increasing TcB. Up to a TcB of approximately 90 μmol/L (D3), the measured TcB was within the manufacturer’s stated accuracy (±25.5 μmol/L) for all mimicked skin colors. In clinical practice, it is common to apply a safety margin for clinical decision making, which is larger (50 µmol/L) than the level of accuracy specified by the manufacturer. Above a TcB of 175 μmol/L (D3), the measured deviation in the TcB exceeded this clinical safety margin for the darker skin colors (L3 and L4). The maximum differences in the measured TcB levels amounted 67–132 μmol/L between the lightest skin color (L0) and more pigmented skin colors (L2–L4) for the highest mimicked TcB (D9: 250 μmol/L). We also observed an increase in the standard deviation of the TcB measurements per phantom with increasing skin color, ranging from 0.77 μmol/L (L0) to 7.3 μmol/L (L4).

## Discussion

The purpose of this study was to evaluate the effect of skin color on the measured TcB. Hereto, we systematically evaluated the TcB meter JM-105 on neonatal skin phantoms in which the mimicked melanin and bilirubin concentrations were varied. We observed a strong dependence of the measured TcB on skin color, with a maximum deviation of 132 µmol/L for the darkest skin color (L4; with a mimicked melanosome volume fraction of 40%) compared to the lightest skin color (L0; with a mimicked melanosome volume fraction of 0%).

The main advantage of using skin phantoms for this purpose, is that we were able to control and quantify skin color, while keeping other properties of neonatal skin constant, such as skin thickness, skin maturity related light scattering, and body location. This is practically impossible in an in vivo patient setting and may explain why current evidence in literature is inconclusive regarding the effect of skin color on TcB levels.^15–24^ In our study, where all other sources of patient variations were excluded except skin color, the TcB was underestimated in darker skin, similar to several other in vivo studies.^17,20–23,25^

It is important to note that we compared all TcB levels to unpigmented skin (L0) in this study, because the TcB manufacturer does not specify for which skin color the TcB meter is calibrated.^14^ If we compare all TcB levels to, for example, slightly more pigmented skin (L1), the underestimation of the dark pigmented range (L2-L4) remains present, but it is reduced by 26 µmol/L, to 41–106 µmol/L (D9: 250 µmol/L). Furthermore, in this study, the melanosome volume fractions in the mimicked epidermal phantom layers were derived from Jacques,^32^ which ranged from 0 to 40% for adult skin. Newborn infants are born with lower melanosome volume fractions in the epidermis compared to adults and this concentration increases during the first six months of life.^35–37^ Although there is no quantitative information available in literature on epidermal melanosome volume fractions in newborn infants, it is reasonable to assume that the highest concentrations that were mimicked in this study are less common in newborn populations.

Additionally, the phantoms in this study are an approximation of neonatal skin, and variations in optical properties and phantom geometry may lead to different results.^26^ Our dermal phantoms mimicked the average amount of light scattering and hemoglobin absorption by neonatal skin and biological variations in these parameters were not taken into account.^34^ A similar argument holds for the thickness and light scattering behavior of the epidermal phantom layer, which was fixed to the average epidermal thickness for newborn skin (39 μm) and a scattering coefficient within the range of various epithelial tissue types (average μ_s_ ≈ 35 mm^−1^).^31^ Furthermore, we mimicked the TcB concentration up to a value of 250 μmol/L, which is above the phototherapy threshold for certain postnatal ages and risk groups.^5,38^ For TcB values > 250 μmol/L, it is likely that the variability between skin colors will further increase. Instead of phantom measurements, Monte Carlo simulations of photon transport in neonatal skin may be used to further unravel the influence of biological variation in patient characteristics. However, this requires knowledge of the exact operating principles of the TcB meter, which remains currently unreported by the manufacturer.

Because of these limitations, it is important to note that a direct translation of our results to clinical practice is challenging on a quantitative basis: the severity of the TcB underestimation in dark skinned patients may differ from the levels presented by our phantom measurements. However, this study does clarify the qualitative effect of skin color on TcB measurements: our phantoms demonstrate with high certainty that darker skin colors lead to larger underestimations of the TcB. These findings only apply to the TcB meter that was evaluated in this study (JM-105) and may be affected by low inter-device reproducibility in other experimental settings.^28^ There are alternative brands of TcB meters available, such as the BiliCare (Mennen Medical Ltd, Israël)^39^; BiliChek (Philips, The Netherlands)^40^; BiliTest (Technomedica, Russia)^41^; AJO-Neo (SNBNCBS, India)^42^; and Mbj20 (Beijing M&B Electronic Instruments, China).^43^ As these other TcB meters employ different approaches to correct for skin color, the protocol that we have introduced here can be directly applied to evaluate these TcB meters as well.

### Clinical implications

This study clarifies the effect of skin color on TcB measurements. This is important for the interpretation of TcB measurements in newborn infants with varying skin colors. Similar to the recently discovered skin color bias in pulse oximetry,^29,30^ our results show that TcB measurements are significantly affected by skin color. Darker skin tones and higher bilirubin levels lead to greater underestimation of TcB. Since the available evidence in literature does not provide a conclusive answer on the impact of skin color on TcB measurements,^15–24^ this study provides more certainty in this regard. Our results highlight the need for improved TcB meter design and cautious interpretation of TcB readings on newborns with dark skin. Clinically, the TcB is used to assess whether a caretaker should be concerned about a child, and whether additional TSB determination is required.^6,38^ Based on the findings of this study, this assessment should also include skin color as a risk factor for underestimating rising bilirubin levels. For darker skin tones, it may be more appropriate to conduct a TSB assessment at lower TcB values. However, we would like to emphasize that further research is necessary to determine the applicability of our findings in the clinical context.

## Conclusion

From this systematic in vitro evaluation, it can be concluded that TcB determinations are significantly affected by skin color. The darker the skin color and the higher the bilirubin level, the greater the underestimation of the measured TcB. For the neonatal skin mimicking phantoms that were used in this study, this underestimation far exceeded the accuracy of the bilirubin meter as specified by the manufacturer and the clinically used safety margin. Our results emphasize the need for i) an improved design of TcB meters that is less biased towards darker skin colors and ii) careful interpretation of TcB measurements in newborns with a dark skin color.

## Data Availability

The data that support the findings of this study are available from the corresponding author, Dam-Vervloet, upon reasonable request.
